# Models of Oral Epithelial Dysplasia: A Systematic Review and Temporal Analysis

**DOI:** 10.1111/jop.70149

**Published:** 2026-05-13

**Authors:** Zilefac Brian Ngokwe, Satutya Wicaksono, Michael McCullough, Antonio Celentano, Tami Yap

**Affiliations:** ^1^ Melbourne Dental School The University of Melbourne Carlton Victoria Australia

**Keywords:** carcinogenesis, in vitro models, in vivo models, oral epithelial dysplasia, precancerous lesions

## Abstract

**Background:**

Oral squamous cell carcinoma (OSCC) is the most common malignancy of the head and neck, associated with substantial morbidity and mortality worldwide. Oral epithelial dysplasia (OED) can precede OSCC, offering a critical window for preventive and therapeutic intervention. This review maps the historical and current landscape of in vitro and in vivo models of OED, providing insight into their strengths, limitations, and translational relevance.

**Methods:**

A systematic review and temporal analysis were conducted following PRISMA guidelines, with literature searches performed across Medline, EMBASE, EBM Reviews, and Web of Science.

**Results:**

From 4009 records, 292 studies from 26 countries were included, yielding 307 models of OED. Only a minority of studies (17.4%) focused primarily on dysplasia as a precancerous condition. In vivo models predominated (88.9%), while in vitro systems were comparatively scarce and largely limited to 2D cultures. Organoid‐based approaches were rarely reported, highlighting a gap in advanced model development. Reproducibility data were available for 125 studies (45.8%). The hamster emerged as the most frequently used animal [*n* = 96, 35%], and 4‐nitroquinoline‐1‐oxide (4‐NQO) remained the most common carcinogen, particularly in murine models. A strong sex bias was observed, with male animals heavily over‐represented.

**Conclusion:**

Overall, models of OED remain under‐represented and under‐developed, particularly in comparison to innovations in other fields of cancer research. Despite the central role of OED in oral carcinogenesis, current models do not adequately reflect clinical diversity or exploit modern 3D and patient‐derived technologies. This review provides a critical reference point to guide future studies toward more accurate, reproducible, and clinically relevant models, with the potential to advance prevention, early detection, and targeted therapies for OSCC.

**Abbreviations**
×/wk: times a week
4‐NQO: 4‐Nitroquinoline 1‐oxide
BQE: betel quid extract
DMBA: 7,12‐Dimethylbenz[a]anthracene
DW: drinking water
F: female
HGD: high grade dysplasia
LGD: low grade dysplasia
M: male
MiD: mild dysplasia
MIR: mortality‐to‐incidence ratio
MoD: moderate dysplasia
NNN: N‐Nitrosonornicotine
NSND: non‐smoking, non‐drinking
OC: oral cancer
OD: oral dysplasia
OED: oral epithelial dysplasia
OHAT: office of health assessment and translation
OPMDs: oral potentially malignant disorders
OSCC: oral squamous cell carcinoma
SD rats: Sprague–Dawley rats
SD: severe dysplasia
Tg mice: transgenic mice
TSNO mice: Tsumura Suzuki non‐obese mice
WHO: World Health Organization

## 1 Introduction

Oral cancer remains a pressing global health challenge, with new oral cancer cases projected to rise by 64.6% by 2050 reaching 641 563 [1] while all cancers are projected to rise by 77% by 2050, reaching 35 million annually [2, 3]. Oral squamous cell carcinoma (OSCC), the most common form, typically arises through a histological continuum from normal epithelium to dysplasia and malignancy [4]. Clinically, this progression is reflected in the World Health Organization's (WHO) classification of oral potentially malignant disorders (OPMDs), with epithelial dysplasia serving as the strongest predictor of malignant transformation [5, 6]. In 2022, oral cancer ranked 16th worldwide for incidence (389 846 cases) and 15th for mortality (188 438 deaths), with a mortality‐to‐incidence ratio (MIR) of 0.48 [7, 8]. The heaviest burdens occur in Asia, largely due to tobacco, alcohol, and betel nut use though underreporting in low‐resource regions may obscure the true scale [9]. Despite decades of research, OSCC outcomes remain poor, with a 5‐year survival at ~50% [10]. Late presentation, treatment toxicity, and limited therapeutic advances contribute to this stagnation. Surgical resection, the current standard of care, carries substantial morbidity, especially for tongue cancers leading to impaired speech, swallowing, and quality of life [11, 12, 13] A critical barrier to progress lies in preclinical modeling. Conventional in vitro and animal systems often fail to reproduce the tumor microenvironment, omitting key features such as stromal interactions, immune infiltration, and molecular dynamics [14]. This translational gap has slowed the development of more effective therapies. With population aging, by 2050 one in six people will be over 65 [15], the OSCC burden will intensify [7, 16]. Intercepting disease at the dysplastic stage provides a unique opportunity: reducing mortality, preventing secondary tumors through field cancerization [17], and alleviating the physical, psychological, and economic toll of advanced disease [18]. Understanding and improving models of oral epithelial dysplasia is therefore vital. This systematic review critically evaluates in vivo and in vitro OED models within the OPMD framework, mapping trends, identifying gaps, and highlighting opportunities for translational advancement.

## 2 Methods

This systematic review followed PRISMA guidelines and was registered on PROSPERO (CRD42025605982). The objective was to identify, map, and critically appraise preclinical models of oral dysplasia within the context of oral potentially malignant disorders.

### 2.1 Search Strategy

A comprehensive search was performed on May 14, 2024, across four databases: MEDLINE (Ovid), EMBASE (Ovid), EBM Reviews (Ovid), and Web of Science. No date or language restrictions were applied. Full search strings are available in the Supporting Information.

### 2.2 Eligibility Criteria

*Studies were eligible if they*:

- Investigated in vivo and in vitro models assessing histologically confirmed oral dysplasia in OPMD contexts.
- Including peer‐reviewed articles, case reports, or conference abstracts

*Exclusion criteria*:

- Studies limited to oral cancer without dysplasia data.
- Clinical studies not describing models of oral dysplasia
- ex vivo, or in silico studies.
- Non‐English publications.

### 2.3 Study Selection

Screening occurred in two stages: (1) title and abstract review, and (2) full‐text assessment. Two reviewers (BN, SW) independently assessed each record; disagreements were resolved by consensus or adjudication with a third reviewer.

### 2.4 Data Extraction

Data were extracted independently by two reviewers (BN, SW) using Covidence. Authors were not contacted for missing data. Extracted items included:

- In vivo *models*: study metadata, animal species/strain/age/sex, carcinogen type/dose/route/frequency, induction period, dysplasia grade, and study aim (precancer vs. cancer).
- In vitro *models*: study metadata, model type (2D vs. 3D), cell source (primary vs. immortalized), dysplasia origin, and study focus.

For studies with multiple time points, data from the timepoint showing highest dysplasia induction was recorded.

Regarding the histopathologic grading of dysplasia, the two reviewers (BN and SW) independently extracted study data. For in vivo studies, histopathologic grading of epithelial dysplasia was recorded as reported in the original articles; where grading terminology varied across studies, the reported classifications were documented verbatim and, where appropriate, mapped to standard categories (mild, moderate, severe) for descriptive synthesis. Finally, the two reviewers carefully verified the grading and cross‐checked it twice to ensure consistency.

### 2.5 Risk of Bias Assessment

Internal validity was assessed using the Office of Health Assessment and Translation (OHAT) risk of bias tool. This OHAT tool permits an evaluation for each study of its design and conduct, based on a structured set of domain‐specific questions designed to evaluate the potential for bias in individual study outcomes. This assessment was conducted independently by two reviewers (BN and SW). In cases of disagreement, a third reviewer was consulted to reach a final consensus.

Eight OHAT domains/questions were applied:

- Selection bias: randomization of exposure/dose.
- Performance bias: comparability of experimental conditions.
- Attrition bias: completeness of outcome data.
- Detection bias: accuracy of exposure and outcome assessments.
- Selective reporting bias: completeness of reported outcomes.

Other biases:

- Appropriateness of statistical analyses.
- Consideration of co‐exposures and confounding factors.

Each item was rated as definitely low, probably low, probably high, or definitely high risk of bias (Supporting Information).

### 2.6 Outcomes

The **primary outcome** was the induction or presence of histologically confirmed oral epithelial dysplasia in a precancerous model.

## 3 Results

### 3.1 Study Selection

**FIGURE 1:**
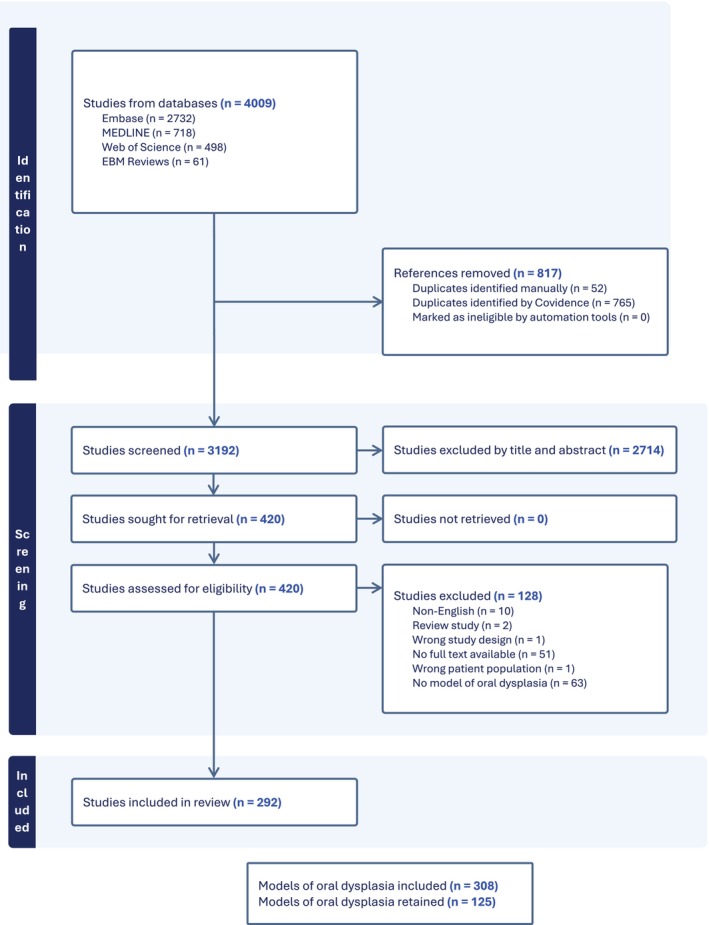
PRISMA chart.

A total of 4009 articles were retrieved across four databases on 14 May 2024: EMBASE (*n* = 2732), MEDLINE (*n* = 718), Web of Science (*n* = 498), and EBM Reviews (*n* = 61). After removing duplicates and applying inclusion and exclusion criteria, 292 studies were eligible for final analysis (Figure 1, PRISMA chart). Among these 292 studies, only 51 (17.5%) specifically aim to develop or characterize oral dysplasia in the context of precancerous lesions.

### 3.2 Model Characteristics

From the 292 studies, we identified 307 individual models of oral dysplasia, as some studies employed multiple models. The majority were in vivo (*n* = 273 (88.9%)) and 34 (11.1%) were in vitro models.

### 3.3 In Vitro Models

Among the 34 in vitro models, 25 (73.5%) were 2D systems, and 9 (26.5%) were 3D tissue‐engineered models.

### 3.4 In Vivo Models

Among the 273 animal models, the species distribution was:

- Hamsters: *n* = 96 (35.2%)
- Mice: *n* = 91 (33.3%)
- Rats: *n* = 87 (31.9%) (Table 1)

**TABLE 1:** Frequency of animal models.

Animal species	Frequency
Hamsters	95
Mice	91
Rats	87
Grand total	273

### 3.5 Geographic Distribution

**FIGURE 2:**
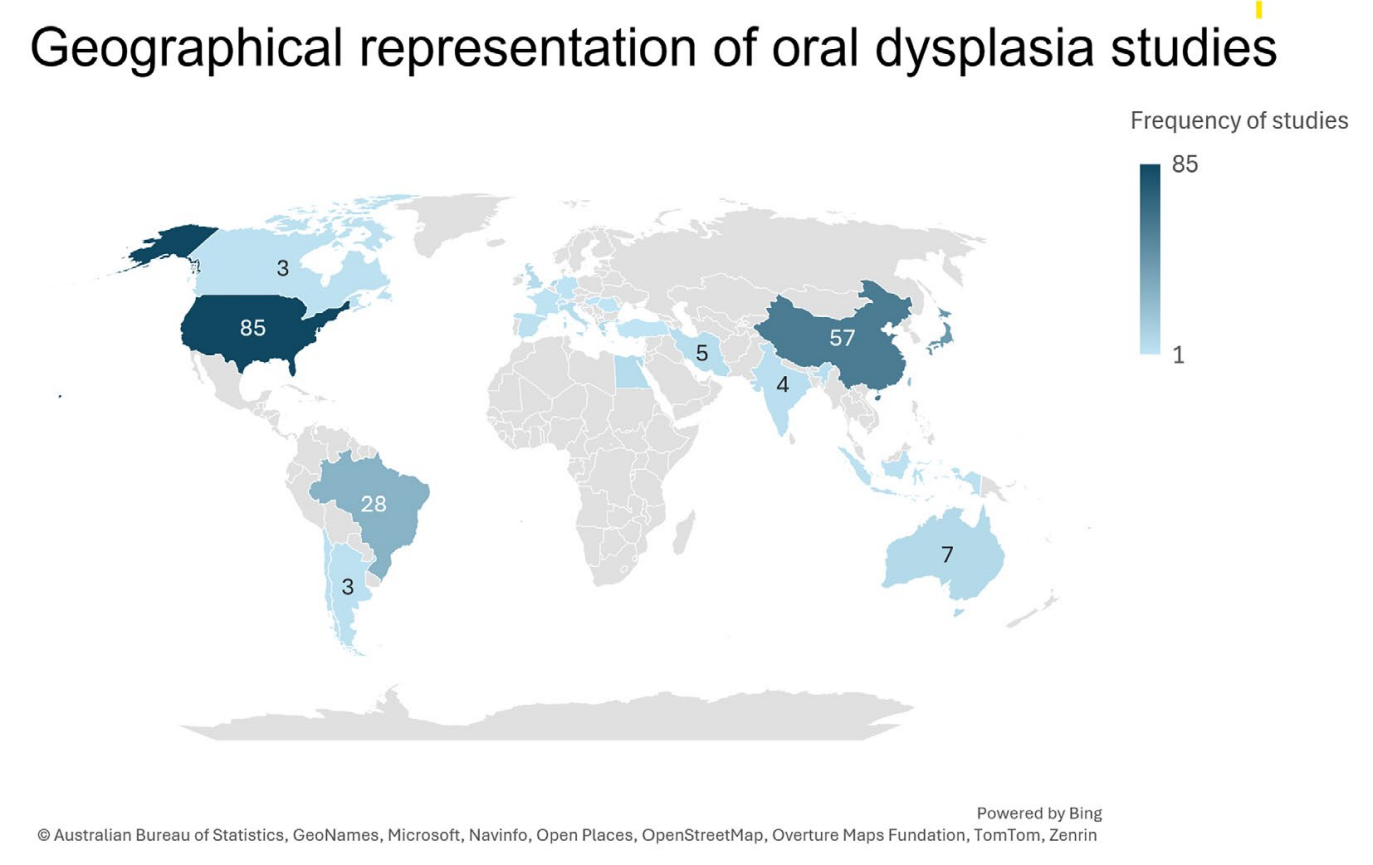
Geographical distribution of oral dysplasia studies.

The studies spanned 26 countries. The most represented were the United States (*n* = 85 models), China (*n* = 57), Japan (*n* = 41), Brazil (*n* = 28), Taiwan (*n* = 20) (Figure 2).

### 3.6 Temporal Trends

**FIGURE 3:**
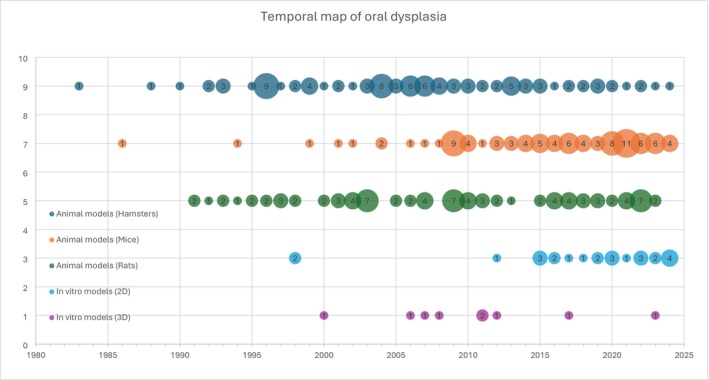
The use of in vitro (2D and 3D) and animal models of OED in documented literature.

Temporal analysis of the 307 models (Figure 3, Temporal Map of oral dysplasia) revealed:

- Animal models have been used consistently since the 1980s, with the earliest model being a hamster study in 1983.
- 3D in vitro models emerged from 2000 onwards.
- Hamster models have declined in frequency over the past decade, while murine models have steadily increased.

### 3.7 Risk of Bias Assessment

**FIGURE 4:**
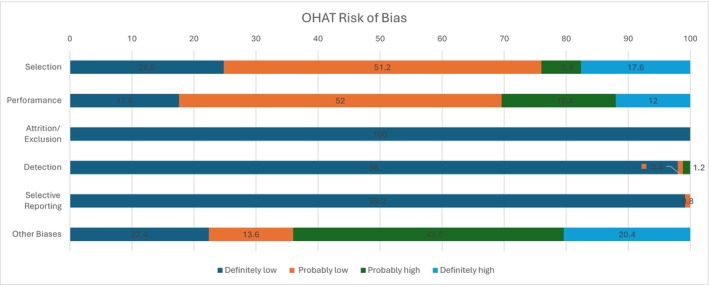
Risk of bias assessment.

Using the OHAT tool, domains of bias were assessed. The risk of bias was “definitely low” for selection (24.8%), performance (17.6%), attrition/exclusion (100%), detection (98%), selective reporting (99.2%), and other domains (22.4%) (Figure 4).

### 3.8 Reproducibility Assessment

To assess reproducibility, we evaluated whether studies reported the key methodological variables necessary to replicate the model, including animal age, sex, weight, carcinogen type, dose, route of administration, frequency of exposure, and time to dysplasia induction. Models were considered reproducible when sufficient detail was provided across these core parameters to allow reliable replication.

A total of 125 models from 119 studies met reproducibility criteria, comprising 91 in vivo models from 90 studies (Table 2) and 34 in vitro models from 32 studies (Table 3). Some studies included both in vivo and in vitro models (Tables 4 and 5).

**TABLE 2:** Distribution of carcinogenic agents in the animal models.

Carcinogenic agent	Animal species			
	Rats	Hamsters	Mice	Total
4‐NQO	36		17	53
DMBA	3	29	1	33
4NQO + arecoline			2	2
N‐Nitrosonornicotine (NNN)		1		1
DMBA and Betel quid extract (BQE)		1		1
Dibenzo[a,l]pyrene (DB[a,l]P)		1		1
Total	39	32	20	91

**TABLE 3:** Distribution of in vitro models.

In vitro models	2D	3D
DOK cell line	18	N/A
Leuk 1 cell line	6	N/A
Leuk 1 s cell line	1	N/A
Organotypic culture	N/A	3
Spheroids	N/A	1
Tissue engineered models	N/A	5
Total	25	9

**TABLE 4:** In vitro 2D models of dysplasia.

First author surname (year)	2 D	Cell type	Primary aim of the study
Yang (2024) [19]	Primary cells	DOK	OD
Shi (2024) [20] Wang (2022) [21] Wang (2019) [22] Wang (2020) [23] Li (2024) [24] Fox (2018) [25] Wang (2017) [26]	Immortalized commercial cell lines	DOK	OD
Kuo (2019) [27] Chen (2020) [28] Li (2022) [29]	Immortalized commercial cell lines	DOK	OD and OC
Lee (2015) [30] Pena‐Oyarzun (2024) [31] Wen (2020) [32] Rajendiran (2015) [33]	Immortalized commercial cell lines	DOK	OC
Hefni (2023) [34] Lan (2016) [35] Lu (2021) [36]	Primary cells	DOK	OC
Lin (2022) [37] Korraah (2012) [38]	Immortalized commercial cell lines	Leuk 1	OD
Anisuzzaman (2016) [39]	Immortalized commercial cell lines	Leuk 1	OC
Hefni (2023) [34]	Primary cells	Leuk 1	OC
Khammanivong (2016) [40] Khafif (1998) [41]	Immortalized commercial cell lines	Leuk 1	OD and OC

**TABLE 5:** In vitro 3D models of dysplasia.

First author surname (year)	3 D	Cell type	Primary aim of the study
Gaballah (2007) [42]	Tissue engineered models	DOK, D6, D20, POE9n	OD
Gaballah (2008) [43]	Tissue engineered models	CDOK, DOK, SPDOK LT1DOK, LT2DOK, POE9n, D6, FM1DOK, FM2DOK, D20	OD
Smith (2011) [44]	Tissue engineered models	D20	OC
Mian (2017) [45]	Tissue engineered models	DOK	OD and OC
Colley (2011) [46]	Tissue engineered models	D20 and DOK	OD and OC
Yoo (2000) [47]	Organotypic culture	Immortal human gingival keratinocyte (IHGK)	OC
Vigneswaran (2006) [48]	Organotypic culture	Leuk 1 and Leuk 2	OC
Dalley (2013) [49]	Organotypic culture	DOK and POE9n	OD and OC
Suryaprakash (2023) [50]	Spheroids	DOK	OC

### 3.9 Detailed In Vitro Results

Amongst the 34 reproducible in vitro models:

- The DOK cell line was the most represented 2D model (*n* = 18, 72%).
- Tissue‐engineered 3D models were the most common among 3D systems (*n* = 5, 55.56%) (Table 3).

### 3.10 Detailed In Vivo Results

Among the 91 reproducible in vivo models:

- Rat were the most prevalent species (*n* = 39, 42.9%).
- The most commonly used carcinogen was 4‐Nitroquinoline 1‐oxide (4‐NQO) (*n* = 53).

Carcinogen use by species:

- Hamsters: predominantly DMBA (*n* = 29, 90.6%)
- Rats: primarily 4‐NQO (*n* = 36, 92.3%)
- Mice: primarily 4‐NQO (*n* = 17, 85%) (Table 2)

Sex distribution:

- Male animals: *n* = 79, (83.5%)
- Female animals: *n* = 9 (9.9%)
- Both sexes: *n* = 6 (6.6%)

Summary of the models:

- In vitro models (*n* = 34): 2D versus 3D systems, including 5 (20%) derived from primary dysplastic tissue and 20 (80%) from immortalized lines (Tables 4 and 5).
- In vivo models (*n* = 91): Ranked by species and carcinogen type, including induction protocols, dysplasia grade, and latency periods to facilitate reproducibility and inter‐study comparison (Table 6).

**TABLE 6:** In vivo studies of dysplasia induction.

First author surname, (year)	Animal species	Animal strain	Animal age (weeks)	Sex	Carcinogenic agent	Dose of carcinogenic agent	Route of application (administration)	Starting weight of animal	Frequency of application (administration)	Dysplasia induction time (weeks)	Dysplasia grade	Primary aim of the study
Li (2013) [51]	Mice	Balb/C	6	M	4‐NQO	20 μg/mL	DW	250 g	N/A	14	MiD	OC
deFaria (2011) [52]	Mice	Tg	4–6	M	4‐NQO	100 μg/mL	DW	21–23 g	N/A	16	N/A	OC
Santos (2014) [53]	Mice	Tg	6	M	4‐NQO	100 μg/ml	DW	21–23 g	N/A	16	MiD to SD	OC
Santos (2017) [54]	Mice	Swiss	4–6	M	4‐NQO	50 μg/mL	DW	20–22 g	N/A	18	N/A	OC
Tanaka (2017) [55]	Mice	TSNO	8	M	4‐NQO	20 ppm	DW	23.6 ± 2.2 g	N/A	28	MoD	OC
deOliveiraSantos (2014) [53]	Mice	Tg	6	M	4‐NQO	100 μg/ml	DW	21–23 g	N/A	16	N/A	OC
Fang (2022) [56]	Mice	C57BL/6	6	F	4‐NQO	50 μg/ml	DW	10–12 g	N/A	16	MiD to SD	OD
Yang (2024) [19]	Mice	C57BL/6	6–8	F	4‐NQO	50 μg/ml	DW	15 ± 2 g	N/A	20	MoD to SD	OD
Czerninski (2009) [57]	Mice	C57Bl/6	4–6	F	4‐NQO	50 μg/mL	DW	18–20 g	N/A	16	N/A	OC
Ottaviani (2016) [58]	Mice	C57BL/6	6	F	4‐NQO	50 μg/ml	DW	20 g	N/A	20	MiD to SD	OC
Neculqueo (2022) [59]	Mice	C57Bl/6	8	F	4‐NQO	50 μg/ml	DW	15–20 g	N/A	16	N/A	OD and OC
Chen (2020) [60] Lu (2021) [36]	Mice	C57BL/6	4–8	M; F	4‐NQO	50 μg/ml	DW	16–30 g	N/A	24	MiD to SD	OC
Kosugi (2019) [61]	Rats	SD	6	M	4‐NQO	50 ppm	DW	260 g	N/A	10	LGD	OC
Schuch (2023) [33] DeMoura (2019) [62] Soares (2018) [60]	Rats	Wistar	8	M	4‐NQO	25–50 ppm	DW	250–300 g	N/A	12	N/A	OC
Sari (2022) [63]	Rats	SD	8–10	M	4‐NQO	30 ppm	DW	180–250 g	N/A	12	N/A	OC
Minicucci (2011) [38] Minicucci (2009) [64] Fracalossi (2011) [65] Fracalossi (2010) [66] Ribeiro (2009) [67] Fracalossi (2010) [68] Noguti (2015) [69]	Rats	Wistar	8	M	4‐NQO	50 ppm	DW	250 g	N/A	12	MiD and MoD	OC
Moon (2012) [70]	Rats	SD	8	M	4‐NQO	50 ppm	DW	250 g	N/A	12	MoD to SD	OC
Vered (2003) [71]	Rats	Wistar‐derived	12	M	4‐NQO	0.001%	DW	200 g	N/A	14	MiD	OC
Vered (2007) [72]	Rats	Wistar	12	M	4‐NQO	0.001%	DW	200 g	N/A	14	MiD to SD	OC
Lv (2017) [73]	Rats	SD	3–4	M	4‐NQO	0.002%	DW	50–75 g	N/A	14–22	N/A	OC
Hong (2007) [74]	Rats	SD	3–4	M	4‐NQO	0.002%	DW	50–75 g	N/A	15	MoD/SD	OC
Neto (2022) [75]	Rats	Rattus nervergicus albinos	4	M	4‐NQO	25 ppm	DW	120–130 g	N/A	16	MoD	OD and OC
Kong (2015) [4]	Rats	SD	8	M	4‐NQO	50 ppm	DW	250 g	N/A	16	N/A	OC
Chen (2018) [76]	Rats	SD	4	M	4‐NQO	20 ppm	DW	75–100 g	N/A	16	N/A	OC
Ohnishi (2016) [77]	Rats	SD	6	M	4‐NQO	50 ppm	DW	200–250 g	N/A	16	SD	OD and OC
Wagner (2021) [17]	Rats	Wistar	8.6	M	4‐NQO	25 ppm	DW	150–200 g	N/A	19	N/A	OC
SPULDARO (2022) [78]	Rats	Wistar	8.6	M	4‐NQO	25 ppm	DW	350 g	N/A	20	N/A	OC
Valente (2018) [79]	Rats	Wistar	12	M	4‐NQO	50 ppm	DW	250–300 g	N/A	20	MiD to SD	OC
Xu (2022) [80]	Rats	Wistar	6–8	M	4‐NQO	40 μg/mL	DW	250–300 g	N/A	24	MiD to SD	OC
DeVisscher (2013) [81]	Rats	Wistar	7	M	4‐NQO	0.001%	DW	200 g	N/A	24	MoD to SD	OD and OC
Okazaki (2002) [82]	Rats	SD	4	M	4‐NQO	50 ppm	DW	200 g	N/A	16	MiD to MoD	OD
Dalal (2022) [83]	Rats	Wistar	6	M	4‐NQO	50 ppm	DW	126–142 g	N/A	20	MiD to MoD	OC
Makita (1997) [84]	Rats	F344	7	M	4‐NQO	20 ppm	DW	347 ± 17 g	N/A	8	MoD to SD	OC
Xia (2009) [85] Xia (2009) [86]	Rats	SD	7	M	4‐NQO	40 ppm	DW	200 g	N/A	9–22	MiD to SD	OC
Ge (2021) [87]	Rats	Wistar	22.9	M	4‐NQO	0.002%	DW	220 ± 10 g	N/A	9	MiD	OC
Kitakawa (2006) [88]	Rats	Wistar	8	M	4‐NQO	50 ppm	DW	250 g	N/A	12	MoD	OC
Hafiz (2020) [89]	Rats	SD	8	F	4‐NQO	20 ppm	DW	219.7 ± 24.9 g	N/A	12	Middle to HGD	OC
Ma (1999) [90]	Mice	CBA	6	M	4‐NQO	0.5%	Topical (palatal)	21–24 g	3×/week	28	N/A	OD and OC
Hawkins (1994) [91]	Mice	CBA	9	M	4‐NQO	5 mg/mL	Topical (palate)	23–27 g	3×/week	24	MiD to SD	OC
Steidler (1986) [92]	Mice	CBA	6.4	M	4‐NQO	0.5%	Topical (palate)	21–24 g	3×/week	2–4	N/A	OC
Schoop (2009) [93]	Mice	CBA	7–8	M	4‐NQO	5 mg/ml	Topical (tongue)	23–27 g	3×/week	24	MiD	OC
Scrobota (2016) [94]	Rats	Wistar	8	M	4‐NQO	0.5%	Topical (oral mucosa)	220 ± 20 g	3×/week	16	MiD to MoD	OC
Nauta (1996) [95]	Rats	Wistar	6	M	4‐NQO	0.5%	Topical (palatal mucosa by painting using a brush)	127 ± 13 g	3×/week	8	N/A	OD and OC
Wang (2022) [21]	Hamsters	Syrian golden	5	M	DMBA	0.5%	Topical (left buccal pouch)	100–120 g	3×/week	10	MoD to SD	OD
Wang (2019) [96]	Hamsters	Syrian golden	7	M	DMBA	0.5%	Topical (left cheek pouch mucosa)	115 g	3×/week	2	MiD	OD
Pourshahidi (2019) [97]	Hamsters	Syrian golden	8	M	DMBA	0.5%	Topical (anterior wall of buccal pouch)	100 g	Every other day	5	MiD	OD
Tsai (2004) [98]	Hamsters	Syrian golden	10–12	M	DMBA	0.5%	Topical (left cheek pouch)	120–150 g	3×/week	8	MoD to SD	OD
Zhou (2006) [99]	Hamsters	Syrian golden	6	M	DMBA	0.5%	Topical (left pouch)	60–80 g	3×/week	24	N/A	OD
Soukos (2001) [100]	Hamsters	Syrian golden	4–6	M	DMBA	0.5%	Topical (right buccal pouch)	80–100 g	3×/week	6	MiD to MoD	OD
Kitakawa (2006) [88]	Hamsters	Syrian golden	6	M	DMBA	0.5%	Topical (left tongue side)	100 g	3×/week	12	MoD	OC
Grawish (2010) [101]	Hamsters	Syrian golden	8.6–12.9	M	DMBA	0.5%	Topical (right buccal pouches)	100–120 g	3×/week	12	MoD to SD	OC
Chen (2005) [102]	Hamsters	Syrian golden	6	M	DMBA	0.5%	Topical (bilateral pouches)	100 g	3×/week	9	N/A	OC
Kim (2004) [103]	Hamsters	Syrian golden	6	M	DMBA	0.5%	Topical (bilateral pouches)	120 g	3×/week	4	MiD	OC
Andrejevic (1996) [104]	Hamsters	Syrian golden	5–6	M	DMBA	0.5%	Topical (inferior frontal mucosal surface of the left buccal pouch)	80–100 g	3×/week	6	MiD	OC
Papakosta (2006) [105]	Hamsters	Syrian golden	5	M	DMBA	0.5%	Topical (left buccal pouch)	100 g	3×/week	10	MiD	OC
Ezzat (2017) [106]	Hamsters	Syrian golden	6	M	DMBA	0.5%	Topical (left buccal pouch)	150–200 g	3×/week	16	MoD	OC
Gomez‐Garcia (2013) [107]	Hamsters	Syrian golden	20	M	DMBA	0.5%	Topical (left cheek mucosa)	134.82 g	3×/week	11	MiD to MoD	OC
Sun (2008) [108]	Hamsters	Syrian golden	6	M	DMBA	0.5%	Topical (left cheek pouch)	60–80 g	3×/week	4	N/A	OC
Zhuang (2021) [109]	Hamsters	Syrian golden	8–10	M	DMBA	0.5%	Topical (left hamster oral pouch)	80–120 g	3×/week	10	SD	OC
Yang (2009) [110]	Hamsters	Syrian golden	6	M	DMBA	0.5%	Topical (left pouch)	60–80 g	3×/week	24	N/A	OC
Schwartz (1996) [111]	Hamsters	Syrian golden	8.6–12.9	M	DMBA	0.5%	Topical (right buccal pouch)	95–125 g	3×/week	14	N/A	OC
Grawish (2008) [112]	Hamsters	Syrian golden	8.6–12.9	M	DMBA	0.5%	Topical (right buccal pouches)	100–120 g	3×/week	7	MoD	OC
Hussein (2022) [113]	Hamsters	Syrian golden	8	M	DMBA	0.5%	Topical (right buccal pouch)	100 g	3×/week	9	MiD	OC
Wang (2015) [114]	Hamsters	Syrian golden	6–8	M	DMBA	0.5%	Topical (left pouch)	100–120 g	3×/week	16	N/A	OC
Lida (1999) [115]	Hamsters	Syrian golden	5	M	DMBA	0.5%	Topical (bilateral buccal pouches)	70–80 g	2×/week	25	N/A	OD and OC
Elbanna (2023) [116]	Hamsters	Syrian golden	5	M	DMBA	0.5%	Topical (left buccal pouch)	80–120 g	3×/week	14	MiD	OD and OC
Mahakian (2014) [117]	Hamsters	Syrian golden	4–5	M	DMBA	2.5%	Topical (right cheek pouch)	150 g	3×/week	5	MiD	OD and OC
Hu (2017) [118]	Hamsters	Syrian golden	5–6	F	DMBA	0.5%	Topical (right oral mucosa)	80–100 g	3×/week	8	LGD	OC
Wang (2024) [119]	Hamsters	Chinese	8	F	DMBA	0.005 μg/L	Topical (bilateral cheek pouch mucosa)	20–25 g	3×/week	15	N/A	OC
Chen (2011) [120]	Hamsters	Syrian golden	6–8	M; F	DMBA	0.5%	Topical (bilateral hamster cheek pouches)	90–120 g	3×/week	6	MiD to MoD	OD
Ricardo (2012) [121]	Hamsters	Syrian golden	12.9	M; F	DMBA	0.5%	Topical (right lateral edge of the tongue)	126.8 ± 14.26 g	3×/week	13	MoD to SD	OC
Raimondi (2005) [122]	Hamsters	Syrian golden	6–7	M; F	DMBA	0.5%	Topical (right cheek pouch)	150–200 g	3×/week	16	N/A	OC
Kasem (2014) [123]	Mice	Egyptian albino	6–8	M	DMBA	0.5%	Topical (dorsal and ventral tongue surfaces)	25 ± 2 g	3×/week	6	N/A	OC
Gkoulioni (2010) [124]	Rats	Wistar	8.6	M	DMBA	0.5%	Topical (left buccal pouch)	350–400 g	3×/week	8	LGD	OD
Maulina (2019) [125]	Rats	SD	8	M	DMBA	0.5%	Topical (buccal mucosa)	200–300 mg	Every two days	4	MiD	OC
Salehi (2017) [126]	Rats	Wistar	7–11	M	DMBA	0.5%	Topical (dorsal surface of the tongue)	160–20 g	Every alternate day	20	MiD to MoD	OD
Chang (2010) [127]	Mice	C57BL/6JNarl	6	M	4‐NQO + arecoline	200 μg/mL 4‐NQO and 500 μg/mL arecoline	DW	21.8 ± 1.5 g	N/A	28	N/A	OC
Chang (2011) [128]	Mice	C57BL/6JNarl	60	M	4‐NQO + arecoline	200 μg/mL 4‐NQO and 500 μg/mL arecoline	DW	22.8 ± 1.6 g	N/A	28	N/A	OC
Schwartz (2004) [129]	Hamsters	Syrian golden	4	F	DB[a,l]P	0.25% DB[a,l]P	Oral gavage down the throat	70–247 g	5×/week	6	MoD to SD	OC
Cheng (2007) [130]	Hamsters	Hamsters	8	M	DMBA and BQE	0.5% DMBA	Topical (buccal pouches)	110–120 g	3×/week	16	N/A	OC
Papageorge (1996) [131]	Hamsters	Syrian golden	8.6	M; F	NNN	40 mg/mL	Topical (unilateral buccal pouch)	100 g	5×/week	24	MiD	OD and OC

Abbreviations: ×/wk, times a week; 4‐NQO, 4‐Nitroquinoline 1‐oxide; BQE, Betel quid extract; DMBA, 7,12‐Dimethylbenz[a]anthracene; DW, Drinking water; F, female; HGD, High grade dysplasia; LGD, Low grade dysplasia; M, male; MiD, Mild dysplasia; MoD, Moderate dysplasia; NNN, N‐Nitrosonornicotine; OC, Oral cancer; OD, Oral dysplasia; SD rats, Sprague–Dawley rats; SD, Severe dysplasia; Tg mice, Transgenic mice; TSNO mice, Tsumura Suzuki Non‐Obese mice.

Due to significant heterogeneity in the study design, methodology, and outcome measures, quantitative analysis or meta‐analyses was not feasible.

We classified this in vitro studies table primarily by the type of model (2D or 3D), each section was further classified by the cell type and finally by the primary outcome of the study (Tables 4 and 5).

### 3.11 In Vivo

We classified this in vivo studies table mainly by carcinogenic agent, then we divided each section by the route of application of the carcinogenic agent. We further subclassified each subgroup by the animal species and then by the sex of the animal.

This division permits us to observe that all studies that used DMBA as a carcinogenic agent used the topical route of application as well as group studies with similar patterns of OED induction as seen in Table 6 below.

Based on our study and to help homogenize future studies we would like to propose a minimum checklist to serve as a guide (Table 7).

**TABLE 7:** Minimum reporting checklist for OED models.

Animal models	In vitro models
Study metadata	Study metadata
Animal species and strain	Type (2D/3D)
Age	Cell line used
Sex	3D (model used)
Weight (starting weight)	Cell source (Primary vs. immortalized)
Carcinogen used (type/dose/route/frequency)	
Time to induce dysplasia	
Dysplasia grade	

## 4 Discussion

Our review highlights a critical gap in oral dysplasia research: only 17.4% of the 292 included studies focused primarily on oral dysplasia, and fewer than half (45.8%) of the 307 identified models were deemed reproducible. This underscores a persistent underappreciation of oral dysplasia as a distinct, clinically relevant entity, rather than merely a transitional stage in oral carcinogenesis.

Oral cancer remains a global health challenge, with marked geographic and demographic disparities. Rising incidence in low‐income countries, women, and individuals under 45 years, particularly among non‐smoking, non‐drinking (NSND) populations—cannot be fully explained by traditional risk factors such as tobacco and alcohol [132, 133, 134]. While increased tobacco and alcohol exposure may explain trends in some populations, they do not fully account for the increase in younger patients or the non‐smoking, non‐drinking (NSND) cohort. This emphasizes the urgent need to identify novel aetiological contributors and implement improved early detection strategies [132].

Oral epithelial dysplasia (OED), as a histological marker of malignant potential, is central to this effort; however, its grading remains subjective, and inconsistent terminology across studies further hampers comparability and translational impact [132, 135, 136, 137].

Globally, the mortality‐to‐incidence ratio (MIR) for oral cancer serves as a surrogate marker of disparities in screening and treatment. With a global MIR of 0.48, Australia stands out with the world's lowest MIR at 0.135 [138, 139]. In contrast, Africa and Asia report significantly higher MIRs (0.71 and 0.57, respectively), reflecting unequal access to early diagnosis and care.

### 4.1 In Vitro Models

Only 11.1% of oral dysplasia models were in vitro, with 2D models (73.5%) dominating over 3D models (26.5%). While 2D cultures have historically facilitated mechanistic studies, they fail to capture the structural complexity, cellular heterogeneity, and dynamic microenvironment of precancerous lesions, limiting their predictive value for translational research [140, 141, 142, 143]. In contrast, 3D systems—including organoids and scaffold‐based constructs—offer a more faithful representation of the tissue architecture and tumor‐stromal interactions critical for understanding dysplasia progression and therapeutic response. Despite the growing adoption of 3D models in oncology, their integration into oral dysplasia research remains minimal: only nine 3D models were identified, eight developed in the last two decades, and none represented patient‐derived organoids. This represents a clear and actionable opportunity to advance the field [143, 144, 145, 146, 147].

A model has to be able to faithfully reflect the human body [148] or tumor in question which it is trying to replicate. With the use of past and current models, there has been no major change to oral cancer mortalities in the last 50 years, so we might need to go back to the drawing board and include models which are closer to the patient and can consistently simulate the human conditions leading to hopefully better outcomes.

With respect to future translational needs, the FDA in its modernization acts 2.0 (2022) and 3.0 (2024) [149] has taken out the need for animal models to validate preclinical studies, paving the way for other preclinical models such as 3D patient derived organoids.

The 2D models and the animal models with their high genetic similarity, [150] lack the genetic diversity found in humans. This lack of genetic variability, accentuated in animal models due to inbreeding and coupled with the differences in how individuals respond to treatment [151] leads to important clinical trial failures [150].

There is no reported patient derived organoid model for OED 17 years after the introduction of PDOs (2009) and 7 years after the first PDO in the head and neck field (2019).

Regarding translational needs, these patient derived organoids as part of the 3D in vitro models seem to offer the best route to validate preclinical studies and then clinical trials which will hopefully lead to validating therapeutic options.

The current model limitations are the lack of 3D models or models that actually replicate the complexity of the OED environment. This leads to the question: can we have a model faithful enough to study, better understand OED? And could better models help diagnose and treat precancers, hence aiding in oral cancer prevention? The ideal next generation model has to be able to recapitulate OED in precancer faithfully, be cost effective, and account for the genetic variation found in humans as well as the complexity of the human systems.

### 4.2 In Vivo Models

Animal models have dominated oral dysplasia research (88.9% of identified models) since the first hamster model in 1983. While these models are invaluable for studying disease progression and therapeutic interventions, they are resource‐intensive, ethically complex, and often limited in translational relevance due to interspecies differences in metabolism, immune response, and carcinogen susceptibility. Our review also highlighted a persistent sex bias, with 83.5% of studies using male animals, which may obscure sex‐specific disease mechanisms and therapeutic responses. 4‐nitroquinoline‐1‐oxide (4‐NQO) emerged as the most commonly used carcinogen (58.2% of in vivo models), reflecting its ability to reliably mimic tobacco‐induced oral carcinogenesis [152, 153]. Yet, despite the reproducibility of animal models, their limited ability to replicate the human precancerous niche may contribute to the persistently low 5‐year survival rate (~50%) for oral cancer. Bridging this gap requires high‐fidelity model systems that incorporate microenvironmental complexity, patient heterogeneity, and early disease biology, particularly for high‐risk and underserved populations such as young patients, NSND individuals, and women.

In terms of limitations to the models. The in vivo models are either fully immune competent or immune deficient and do not represent partial, drug‐induced or age related immune‐suppression which could be a bias in comparison to the human system. Another possible limitation to our study is that, throughout, we did not consider any immunological endpoints as it was not part of our objectives.

Regarding in vitro models, we have more established cell lines for OSCC than for OED. This could be due to the unstable nature of the OED cell lines, which could also often differentiate or revert to normal cells in culture.

Finally, OPMDs do not have a straightforward molecular trajectory; in addition, clinical entities such as PVL (proliferative verrucous leukoplakia) and erythroplakia have a higher transformation risk as well as differentiated dysplasia. This heterogeneity and molecular variability have to be accounted for by optimal models and hence aid in oral cancer prevention.

A summary of the advantages and limitations of the in vivo and in vitro models can be found below (Table 8).

**TABLE 8:** Advantages and limitations of in vivo and in vitro models.

Models		Advantages	Limitations
In vivo		Presence of an Immune system	Expensive
		Uncomplicated genetic engineering	Lack of heterogeneity
		Inter‐organ communication	Lack of genetic variability
			Ethical concerns/animal rights
			Low correlation with clinical trials
In vitro	2D	High throughput	Oversimplistic model
		Cheap	Lack of tumor recapitulation
		Fast	
	3D	Can include tumor heterogeneity	Not ideal for high throughout as compared to 2D
		Faithful and genetically similar	Relatively low success rate
		Can faithfully recapitulate the tumor	
		Proficient drug testing	

## 5 Conclusion

Our findings reveal that oral dysplasia models are inconsistently described and poorly characterized, with a heavy reliance on animal models and underutilization of advanced in vitro systems. To accelerate translational research and improve clinical outcomes, there is a pressing need to adopt high‐fidelity models, particularly 3D cultures and patient‐derived organoids, which better recapitulate the human oral precancerous niche.

Advancing the modeling of oral dysplasia within oral potentially malignant disorders (OPMDs) will not only improve mechanistic understanding but also facilitate early diagnosis, risk stratification, and preclinical testing of preventive and therapeutic strategies. By aligning model development with the unmet clinical challenges—especially among vulnerable populations and regions with high mortality—research can meaningfully reduce the global burden of oral cancer.

## Ethics Statement

The authors have nothing to report.

## Conflicts of Interest

The authors declare no conflicts of interest.

## Supporting information


**Data S1:** MEDLINE (Ovid), EMBASE (Ovid), EBM reviews (Ovid), and Web of Science.

**Data S2:** OHAT risk of bias assessment 2.

## Data Availability

The data that supports the findings of this study are available in the supporting information of this article.
