# Supplementary material for: Models of Oral Epithelial Dysplasia: A Systematic Review and Temporal Analysis
Source: J Oral Pathol Med. 2026 May 13;55(7):755–73. doi: 10.1111/jop.70149 (PMC13429376; doi:10.1111/jop.70149)
Supplement: Supplementary file 1 — Data S1: MEDLINE (Ovid), EMBASE (Ovid), EBM reviews (Ovid), and Web of Science. [file JOP-55-755-s002.docx]

**MEDLINE (Ovid), EMBASE (Ovid), EBM Reviews (Ovid), and Web of Science**

| Search # | Query |
| --- | --- |
| 1 | (oral or mouth* or tongue or floor or palate or lingual or buccal or lip or labial* or mucosa* or retromolar or cheek or gingiva or intra-oral or vermillion border*).mp. |
| 2 | (pre-cancer* or dysplasia or oral epithelial dysplasia* or Leukoplakia or white patches or erythroplakia or red patches or erythroleukoplakia or precancer* or oral potentially malignant disorder or oral potentially malignant lesion or proliferative verrucous leukoplakia or dysplasia or premalignant or pre-malignant or lichen planus or actinic cheilitis*).mp. |
| 3 | (animal model* or murine model* or mouse model* or organotypic culture* or organoid* or spheroid* or tissue-engineer* or tissue engineer* or in vivo model* or in vitro model* or 2D cell cultures* or organ-on-chip* or biomimetic* or tissue equivalent*).mp. |
| 4 | 1 and 2 and 3 |
